# Tetraspanin-induced death of myeloma cell lines is autophagic and involves increased UPR signalling

**DOI:** 10.1038/sj.bjc.6605291

**Published:** 2009-09-15

**Authors:** V Zismanov, M Lishner, S Tartakover-Matalon, J Radnay, H Shapiro, L Drucker

**Affiliations:** 1 Oncogenetic Laboratory, Meir Medical Center, Kfar Saba 44281, Israel; 2 Sackler Faculty of Medicine, Tel Aviv University, Tel Aviv 69978, Israel; 3 Department of Internal Medicine, Meir Medical Center, Kfar Saba 44281, Israel; 4 Hematological Laboratory, Meir Medical Center, Kfar Saba 44281, Israel

**Keywords:** CD81, CD82, tetraspanins, ER-stress, autophagy, multiple myeloma

## Abstract

**Background:**

Multiple myeloma (MM) therapy is hindered by the interaction of the heterogeneous malignant plasma cells with their microenvironment and evolving drug resistance. We have previously shown that the membranal tetraspanins, CD81 and CD82, are under-expressed in MM cells and that their reintroduction causes massive non-apoptotic death. In this study, we aimed to characterise the tetraspanin-induced MM death.

**Methods:**

Multiple myeloma cell lines were transiently transfected with eGFP–CD81N1/CD82N1 fusion proteins and assessed for death mode by flow cytometry (propidium iodide, ZVAD-fmk, 3MA), activation of unfolded protein response (UPR), and autophagy (immunoblot, RT–PCR).

**Results:**

Cell death induced by CD81N1 and CD82N1 in MM cell lines was autophagic and involved endoplasmic reticulum (ER)-stress manifested by activation of UPR pathways, PERK (protein kinase-like ER kinase) and IRE1 (inositol-requiring 1). We also established the relative X-box binding protein 1 baseline expression levels in a panel of MM cell lines and their general dependence on autophagy for survival. Timeline of UPR cascades and cell fate supported our results.

**Interpretation:**

This is the first publication implicating tetraspanins in UPR signalling pathways, autophagy, and autophagic death. Integration of our findings with published data highlights the unifying dependence of MM cells on ER–Golgi homoeostasis, and underscores the potential of tetraspanin complexes and ER-stress as leverage for MM therapy.

Multiple myeloma (MM) is an incurable malignancy of end-stage B lymphocytes (plasma cells). Effective disease treatment is hampered by the complex interactions of the cells with the bone marrow microenvironment and difficulties in defining genes that are integral to the malignant phenotype (Anderson, 2007). It is accepted that effective MM treatment will need to address the cells in context of their supportive microenvironment, as well as target compound signalling cascades to overcome cell heterogeneity and evolving resistance (Anderson, 2007). A unifying and unique feature of plasma and MM cells is their extensive protein synthesis and concomitant cellular adaptations (Barnhart and Simon, 2007).

Protein synthesis, maturation, assembly, and delivery are executed in the endoplasmic reticulum (ER) and Golgi apparatus. Multiple myeloma cells are characterised by expanded ER (Cenci and Sitia, 2007). Misfolded proteins in the ER are directed to the proteasome for degradation (ER-associated degradation – ERAD) (Ding *et al*, 2007). Insufficient ERAD causes proteins to accumulate in the ER, induces ER-stress, and activates signalling cascades. This series of events is termed the unfolded protein response (UPR). The UPR consists of three major pathways initiated by GRP78/BiP activation of ER-stress sensors (activating transcription factor (ATF6), high inositol-requiring 1 (IRE1), and double-stranded RNA-activated protein kinase-like ER kinase (PERK)), and ending in transcriptional modifications that promote the ER capacity for effective protein folding (volume and chaperones) and diminish the protein synthesis rate (Ron and Walter, 2007). In fact, recent studies have showed that elevation of the IRE1-induced transcription factor, X-box-binding protein 1 (XBP1), is essential for terminal B-cell maturation and that an animal model overexpressing XBP1 developed MM (Reimold *et al*, 2001; Carrasco *et al*, 2007). It was also shown in two MM cell lines that partial UPR is constitutively activated (Patterson *et al*, 2008).

Unfolded protein response activation, while having the capacity to rescue cells by providing a window of opportunity to overcome the stress source, can also result in cell death when the stress is not resolved (prolonged and/or too severe) (Ron and Walter, 2007). The ensuing cell death can be apoptotic, necrotic, autophagic, or a combination of these. Crosstalk between autophagy and apoptosis exists as well, resulting in mutual dependence and some redundancy (Gozuacik and Kimchi, 2007).

Autophagy is a highly regulated process usually activated in response to adverse environment during which cytoplasmic materials are enclosed in double membrane-bound vesicles (autophagosomes) that are then targeted by the lysosome for degradation. It is an essential process that allows cell conservation under stress conditions such as nutrient deprivation, and is implicated in both cell death and survival (Ding *et al*, 2007).

Previously, we showed that MM cells and cell lines underexpress tetraspanin members, CD81 and CD82, compared with normal plasma and peripheral blood B cells (Tohami *et al*, 2004, 2007; Drucker *et al*, 2006). Tetraspanin proteins facilitate the spatial organisation and localisation of multi-protein complexes in distinct membranal microdomains that are important to exterior–interior cell signalling. As molecular coordinators, the tetraspanins are involved in many fundamental biological pathways and are correlated with the malignant process and prognosis (Lazo, 2007). The importance of CD81 and CD82 to MM was shown in a study that reintroduced these tetraspanins in fusion vectors with eGFP (CD81N1, CD82N1) into MM cell lines. The tetraspanin overexpression resulted in significant cell death, described as primarily necrotic and caspase-independent (Tohami *et al*, 2007).

In the current study, we aimed to characterise further, the mode of CD81N1/CD82N1-induced MM death and to delineate the signals and constituents that are influenced by tetraspanins. Our major findings showed that the tetraspanins caused ER-stress, UPR activation, and autophagic cell death. This is the first publication implicating tetraspanins in autophagic signalling pathways and autophagic death. Furthermore, our findings underscore the potential of tetraspanin complexes, ER-stress, and their combination as possible means for effective MM therapy.

## Materials and methods

### Cell lines

Multiple myeloma cell lines RPMI 8226 and U266, purchased from the American Type Culture Collection (ATCC, Manassas, VA, USA), and ARP1, ARK, and CAG (provided by Professor Epstein (Little Rock, AR, USA)) were cultured in RPMI 1640 supplemented with 20% heat-inactivated fetal bovine serum (FBS) and antibiotics (Biological Industries, Kibbutz Beit Haemek, Israel). PC3 and Jurkat cell lines (human prostate cancer and T-cell Leukaemia, respectively) were provided by Collgard Company (Petach Tikva, Israel) and cultured in RPMI 1640 supplemented with 10% FBS. Jurkat cells were also supplemented with sodium pyruvate, HEPES buffer, and nonessential amino acids (Biological Industries).

### Transient transfection

Purified plasmids pEGFP-N1 (mock), CD81N1, and CD82N1 were separately introduced into RPMI 8226 and CAG cells as described previously (Tohami *et al*, 2007). Fluorescence (⩾10 000 events per analysis) was analysed by Coulter Flow Cytometer (FACS) (EPICS-XL, Beckman Coulter Inc., Fullerton, CA, USA) as detailed previously (Tohami *et al*, 2007).

### Flow cytometry

#### Cell survival

Transfected and untransfected cells were harvested 18, 24, or 48-h post-treatment/transfection and stained with 1 *μ*g ml^−1^ propidium iodide (PI) for 10 min. PI^−^/PI^+^ cells were enumerated by FACS. Propidium iodide negative or eGFP^+^/PI^−^ were considered the surviving cell fraction (untransfected and transfected, respectively), whereas the PI^+^ and eGFP^+^/PI^+^ were referred to as the fraction of dead cells (untransfected and transfected, respectively).

#### Cell death mode

RPMI 8226 and CAG cells were incubated with 0.05 mM pan-caspase inhibitor, ZVAD-fmk (R&D systems, Minneapolis, MN, USA), and/or 10 mM autophagy formation inhibitor, 3-methyladenine (3MA) (Sigma, Rehovot, Israel), an inhibitor of the class III phosphatidylinositol 3-kinase (Stroikin *et al*, 2004), 20 *μ*M JNK inhibitor, SP600125 (Biomol Int, Plymouth Meeting, PA, USA), or 10 *μ*M MEK1/2/ERK signalling inhibitor, U0126 (Cell Signaling Technology, Danvers, MA, USA), (all dissolved in DMSO) 4 hours after 81N1 and 82N1 transfection. Cell survival (eGFP^+^/PI^–^ cells) was determined 18, 24, or 48 h post-transfection by flow cytometry. Untreated 81N1- and 82N1-transfected cells were considered as controls.

### Cell sorting

Transiently transfected cells were harvested 18 or 24 h post-transfection and passed several times through a syringe for clump dispersion. Next, eGFP^+^ and eGFP^−^ cells (5 × 10^6^ cells per ml in PBS supplemented with 10% FBS) were isolated and collected using a BD FACSAria cell sorter (BD Biosciences, Sparks, MD, USA). Matching cells treated with respective transfection reagent only, were considered eGFP^−^ and used for calibration of eGFP^+^ cell threshold.

### Western blotting

eGFP^+^-sorted cells were lysed in a lysis buffer containing 50 mM HEPES, 150 mM NaCl, 1% Triton X-100, 0.1% SDS, 50 mM NaF, 10 mM NaPPi, 2 mM NaVO_3_, 10 mM EDTA, 2 mM EGTA, 1 mM PMSF, and 10 *μ*g ml^−1^ Leupeptin, for 10 min on ice. The autophagosome-related MAP LC3 protein (LC3I and its modified form LC3II) were detected with a rabbit polyclonal antibody (1 : 2000 dilution, Sigma). Rabbit anti-pJNK (Thr183/Tyr185), total JNK, pmTOR (Ser2448), total mTOR, Beclin 1, and GRP78/BiP (1 : 1000 dilution) were all from Cell Signaling Technology. Rabbit anti-GADD34 (1 : 1000) and GADD153/CHOP (1 : 500) were from Santa Cruz (Santa Cruz, CA, USA). Protein samples of 300 000 cells each were mixed 1 : 5 with loading buffer, separated by SDS–PAGE, and transferred to a nitrocellulose/PVDF membrane. After blocking non-specific binding sites with 5% milk powder, membranes were treated with primary antibodies at 4°C overnight. Bound antibodies were visualised using peroxidase-conjugated secondary antibody (1 : 10 000; Jackson ImmunoResearch Laboratories, West Grove, PA, USA; 75 min at room temperature), followed by enhanced chemiluminescence (ECL) detection (ECL kit, Santa Cruz or Pierce, Rockford, IL, USA). Products were visualised with LAS3000 Imager (Fujifilm, Greenwood, SC, USA). Integrated optical densities of the immunoreactive protein bands were measured as arbitrary units employing Multi Gauge software (Fujifilm).

### Reverse transcription polymerase chain reaction

Total RNA was extracted from 18, 24, or 48-h transfected cells with Purescript (Gentra Systems, Minneapolis, MN, USA). Total RNA (1 *μ*g) was reverse transcribed (Reverse-iT 1st strand synthesis kit, ABgene, Epsom, UK) and amplified for XBP1/XBP1s and housekeeping *β*-actin (3 and 1.5 *μ*l cDNA, respectively). The PCR was optimised at 94°C for 2 min, followed by 38 cycles of 15 s at 94°C, 60 s at 60°C, and 30 s at 72°C, using 4 pmol XBP1/XBP1s primers (F, 5′-CCTTGTAGTTGAGAACCAGG-3′ and R, 5′-GGGGCTTGGTATATATGTGG-3′) and 2 pmol *β*-actin primers (F, 5′-GAGACCTTCAACACCCCAGC-3′ and R, 5′-GCTCATTGCCAATGGTGATG-3′). Products were electrophoresed on 2.2% agarose gels stained with ethidium bromide and visualised with Gel Doc 2000 and Multi-analyst software (Bio-Rad, Hercules, CA, USA). Amplification products of XBP1 observed in gels included the spliced, unspliced, and hybrid (duplex of full-length and spliced) forms (Lin *et al*, 2007). Spliced XBP1 was calculated from the sum of quantified XBP1s band and half the quantity of the hybrid form; total XBP1 was deduced from the combined sum of all bands. X-box binding protein 1/XBP1s expression was normalised to respective *β*-actin. Average expression of all myeloma and non-myeloma cell lines was calculated (arbitrary units) and statistically compared. CD81N1/CD82N1 samples were compared with mock (N1)-transfected controls and ratios were expressed as fold change.

### Statistical analysis

Paired Student's *t*-tests were used to analyse differences between cohorts. A *P*-value of less than or equal to 0.05 was considered significant. An antagonistic effect was verified by drugs’ interaction formula *q*=P(A+B)/P(A)+P(B)−P(A) × P(B) (*q*<0.85− antagonist; *q*>1.15 – synergist; 1.15>*q*>0.85 – additive) (Su *et al*, 2004) assuming that tetraspanin's transfection is the first treatment (A) and 3MA treatment is the second (B). All experiments were repeated separately three to seven times.

## Results

Previously we have reported that reintroduction of the tetraspanins, CD81N1 and CD82N1, into CAG and RPMI 8226 MM cell lines resulted in significant non-apoptotic cell death compared with mock-transfected cells (Tohami *et al*, 2007). Acknowledging the necessity of MM cells to incur changes that support the intensive protein synthesis and ER–Golgi equilibrium typical to them, we hypothesised that the tetraspanins’ effect may involve an autophagic form of cell death due to ER-stress and UPR activation. The relevance of our hypothesis was preliminarily tested by addressing the activation of UPR and autophagy in MM cell lines’ panel (untransfected).

### Autophagy facilitates basal MM cell lines survival

**Figure 1:**
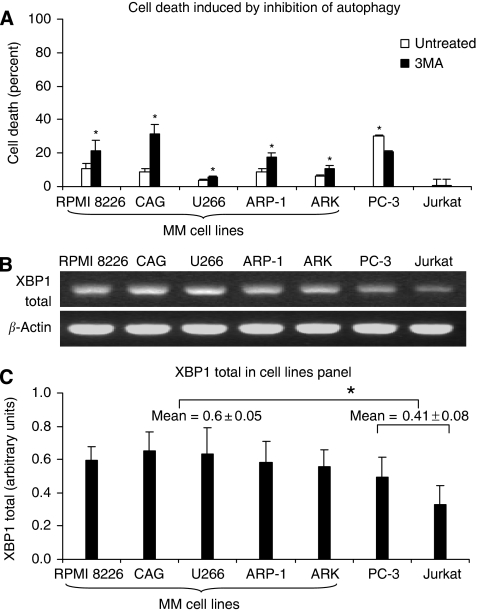
Multiple myeloma cell lines’ basal levels of total XBP1 and dependence on autophagy. (**A**) Cancer cells lines (x-axis) treated with/without autophagy inhibitor 3MA (10 mM) for 24 h were assessed for cell death by FACS (10 000 events) employing PI exclusion assay. Propidium iodide negative cells were considered viable and PI^+^ were regarded as dead cells. Results are expressed as mean proportion of PI^+^ cells per total cell count±s.e. (y-axis) of at least four separate experiments. Statistically significant differences (*P*<0.05) are indicated by ^*^. (**B**) An exemplary picture of XBP1 and *β*-actin (depicted on the left) semi-quantitative RT–PCR conducted on a panel of cell lines (designated on top) and separated by electrophoresis on a 2.2% agarose gel stained with ethidium bromide. (**C**) Graphic presentation of total XBP1 in cancer cell lines (x-axis). Semi-quantitative RT–PCR of total XBP1 was normalised to housekeeping *β*-actin in each sample. Results are expressed as mean±s.e. of at least three separate experiments. Statistically significant differences (*P*<0.05) are indicated by ^*^.

We assessed the importance of autophagy to MM cell lines homoeostasis. We found that inhibiting autophagosome formation using 3MA resulted in elevated levels of cell death (up to 10%, *P*<0.05) in all MM cell lines (Figure 1A), but not in the prostate cancer and T-cell leukaemia cell lines (PC3 and Jurkat, respectively).

We also determined XBP1 transcript level in MM cell lines and established a relative baseline for further reference (Figure 1B and C). All myeloma cell lines express detectable levels of total XBP1 (mean=0.6) that are significantly higher than non-myeloma cell lines (PC3, Jurkat) (mean=0.41, *P*<0.05), which is in agreement with published data (Carrasco *et al*, 2007; Patterson *et al*, 2008).

The connection between ER-stress and activation of UPR and the induction of autophagy is well established, yet autophagy may function as a means of cell preservation as well as a mechanism of cell death (Ogata *et al*, 2006; Ding *et al*, 2007). Moreover, it was shown that increased autophagy levels in yeast facilitate the removal of excess ER after UPR activation, thus promoting ER-homeostasis (Kincaid and Cooper, 2007). Taken together, our results indicate that MM cell lines display a general dependency on autophagy for survival, which may be maintained through a basal condition of ER-stress and activation of UPR signalling cascades.

### Autophagic death induced by CD81N1 and CD82N1 in MM cell lines

**Figure 2:**
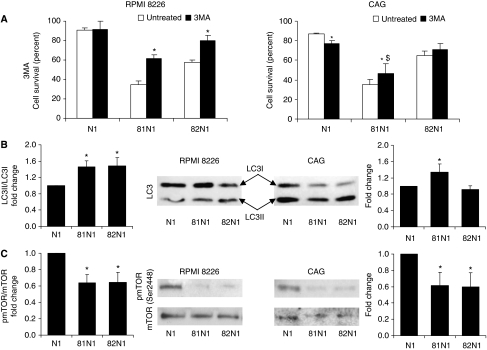
CD81N1/CD82N1 induce autophagic cell death in MM cell lines. RPMI 8226 and CAG cells (depicted at the top) were transiently transfected with N1 (mock), CD81N1, or CD82N1 (x-axis) and assessed for autophagy death mode. (**A**) Transfected cell lines RPMI 8226 (48 h post-transfection) and CAG (24 h) with/without autophagy inhibitor 3MA (10 mM) were assessed for survival by FACS. Results are calculated as the proportion of surviving transfected cells (eGFP^+^/PI^−^) in each sample and expressed as the mean relative percentage±s.e. compared with total eGFP^+^ mock-transfected cells. Statistically significant (*P*<0.05) differences are indicated as ^*^, antagonistic changes indicated by $ (*q*<0.85). At least three separate experiments in duplicate were conducted and FACS recorded 10 000 events. (**B**) Representative immunoblot (300 000 cells per lane) of LC3 (I and II depicted by arrows in central panel) and densitometry of at least three separate experiments are presented (mean±s.e.). LC3II/LC3I were normalised to mock N1 and expressed as fold change. Statistically significant differences (^*^P<0.05) are depicted. (**C**) Representative immunoblot of mTOR and pmTOR (Ser2448) (central panel) (300 000 cells per lane) and densitometry of at least three separate experiments are presented (mean±s.e.). pmTOR/mTOR were normalised to mock N1 and expressed as fold change. Statistically significant differences (^*^P<0.05) are depicted.

Next, we examined the mode of cell death induced by CD81N1/CD82N1 in RPMI 8226 and CAG cells. Transfected cells supplemented with 3MA displayed a significant ∼25% (*P*<0.05) rescue from cell death of CD81N1- and CD82N1-transfected RPMI 8226 cells (48 h) and 12% rescue in CD81N1-transfected CAG cells (24 h) (*P*<0.05, *q*<0.85) compared with transfected cells not supplemented with 3MA (Figure 2A). In addition, a significant elevation in the proportion of LC3II (*vs* LC3I) compared with the mock control was observed in CD81N1/CD82N1-transfected RPMI 8226 (24 h) and CD81N1-transfected CAG cells (18 h) (35–45%, *P*<0.05, Figure 2B). Our results also display significant increases in absolute LC3II levels (not relative to LC3I) compared with mock-transfected cells in CD81N1-transfected RPMI 8226 (38%) and CAG cells (60%), and in CD82N1-transfected RPMI 8226 cells (34%) (*P*<0.05), an analysis method suggested to be more reliable for determining autophagy (Mizushima and Yoshimori, 2007). It should be noted that CD82N1-transfected CAG cells did not display an increase in LC3II (absolute and relative), nor did they respond to 3MA compared with the mock control, all in accordance with a non-autophagic cell death mode (Figure 2A and B). Next, we co-administered the caspase inhibitor, ZVAD, to tetraspanin-transfected MM cell lines treated with 3MA (data not shown). The combined application of 3MA and ZVAD did not differ from CD81N1/CD82N1-transfected cells treated with 3MA alone, indicating that there was no shift in the death mode of the MM cell lines when the autophagy and/or apoptotic cascades were blocked.

For additional validation of autophagic modulation, we examined levels of the established inhibitor of autophagy, mTOR, which normally converts metabolic and mitogenic signals into protein synthesis, and is a recognised target in MM therapeutics (Hu *et al*, 2003). Assessment of phosphorylated and total mTOR levels in CD81N1/CD82N1-transfected MM cell lines displayed decreased levels of active mTOR compared with mock-transfected control (24 h post-transfection, 35–40%, *P*<0.05), which is in sync with autophagic activation (Figure 2C). These findings are also in accordance with previous results that described a decrease in the number of tetraspanin-transfected MM cells expressing pmTOR (Lishner *et al*, 2008).

**Figure 3:**
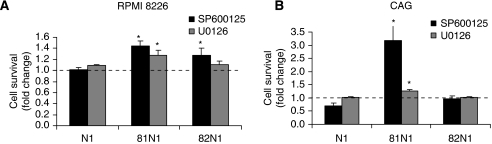
Involvement of ERK and JNK in CD81N1/CD82N1-induced MM cell death. Cell survival of CD81N1/CD82N1 transiently transfected RPMI 8226 (**A**) and CAG (**B**) cells with/without 20 *μ*M JNK inhibitor SP600125 (48 and 24 h post-transfection, respectively) or 10 *μ*M MEK1/2/ERK signalling inhibitor U0126 (24 h) was measured by FACS. Results are calculated as the proportion of surviving transfected (eGFP^+^/PI^−^) cells in each sample and expressed as the mean relative percentage±s.e. compared with total eGFP^+^ mock-transfected cells. N1/81N1/82N1 transfections with treatment (SP600125 or U0126) were normalised to untreated transfection and expressed as fold change. Statistically significant differences (^*^P<0.05) are indicated. At least three separate experiments in duplicate were conducted, and FACS recorded 10 000 events.

Next, we assessed cellular levels of Beclin 1, an established component of the autophagic machinery (Cao and Klionsky, 2007), in the tetraspanin *vs* mock-transfected MM cell lines, but failed to determine significant changes (data not shown). Interestingly, several recent publications present evidence of a ‘Beclin-independent autophagic pathway’ distinguished with an ERK and/or JNK-induced autophagy (Chu *et al*, 2007; Dagda *et al*, 2008). Therefore, we examined the importance of ERK and JNK signalling to transfected MM cells. Inhibition of MEK/ERK resulted in increased cell survival of CD81N1-transfected RPMI 8226 and CAG cells (24 h post-transfection, ∼1.26-fold change, *P*<0.05) as did inhibition of JNK, which resulted in increased survival of CD81N1-transfected RPMI 8226 (48 h) and CAG cells (24 h) (1.45- and 3.18-fold change, respectively; *P*<0.05) and of CD82N1-transfected RPMI 8226 cells (24 h, 1.27-fold change; *P*<0.05) (Figure 3). The involvement of JNK in the death of CD81N1-transfected CAG cells was reported by us previously in association with activation of Forkhead box O (FoxO) (Lishner *et al*, 2008).

### Increased ER-stress and UPR signalling in CD81N1/CD82N1-transfected MM cell lines

**Figure 4:**
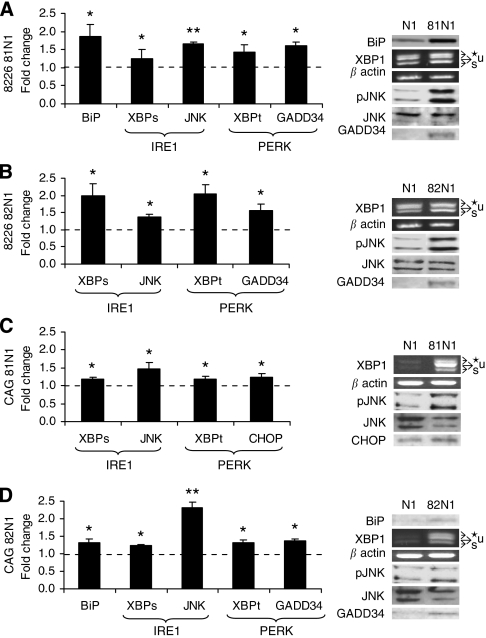
Activated UPR signalling in transfected MM cell lines. RPMI 8226 and CAG cells were transfected with N1, CD81N1, and CD82N1 and assayed 18/24 h later (detailed in Result section). Unfolded protein response components were detected by immunoblot (BiP, JNK, GADD34, CHOP) or semi-quantitative RT–PCR (XBP spliced (XBPs) and XBP total (XBPt)). Graphic presentations (mean±s.e.) of densitometry (*N*⩾3) of RPMI 8226 81N1 (**A**) 82N1 (**B**), and CAG 81N1 (**C**) 82N1 (**D**) (indicated on the left of each panel) cells are presented. Representative immunoblots (300 000 cells per lane) and an exemplary picture of XBP1 RT–PCR are presented in the right side of each panel. BiP, pJNK/JNK, GADD34, and CHOP were normalised to mock N1 and expressed as fold change. Arrows in XBP RT–PCR picture depict the unspliced (u), spliced (s) and hybrid (★) forms. Expression levels were normalised to *β*-actin, fold changes were calculated in total and spliced XBP1 levels relative to mock and expressed as mean±s.e. of at least four separate experiments. Statistically significant differences (^*^P<0.05, ^**^P<0.01) are depicted.

Given that autophagy may be activated by ER-stress, we decided to examine whether this was the case in our research model. Analysis of UPR signalling in the tetraspanin-transfected MM cell lines showed a common activation. We assessed levels of the ER-stress sensor, GRP78/BiP, the IRE1 pathway components, XBP1s and pJNK (Thr183/Tyr185), and the PERK pathway components, XBPt, GADD34, and GADD153/CHOP (Ron and Walter, 2007). Significant changes indicative of IRE1 and PERK activation compared with mock-transfected control were determined in both CD81N1- and CD82N1-transfected RPMI 8226 (24 h post-transfection) and CAG cells (18 h post-transfection except GADD34 and CHOP that were detected 24 h post-transfection) (Figure 4).

Specifically, in CD81N1-transfected RPMI 8226 and CD82N1-transfected CAG cells, increased BiP was detected (88 and 32%, respectively; *P*<0.05). Increased XBPs (20–100%) and pJNKs (37–230%) were shown using both tetraspanin vectors in RPMI 8226 and CAG cells (*P*<0.05). Increased levels of XBP1t (30–200%) and GADD34 (37–56%) were shown in CD81N1-transfected RPMI 8226 cells and in both cell lines transfected with CD82N1 (*P*<0.05). Increased levels of XBP1t (19%) and CHOP (25%) were determined in CD81N1-transfected CAG cells (*P*<0.05).

In summary (Table 1), these results show several facets of UPR activation and, therefore, substantiate increased ER-stress in the tetraspanin-transfected MM cell lines. The consequential cell death was mostly autophagic except for the CD82N1-transfected CAG cells, which display ER-stress, yet their death mode is necrotic (by elimination: not autophagic (Figure 2) and not apoptotic (Tohami *et al*, 2007)).

### Timeline of tetraspanin-induced UPR and cell death in MM cell lines

**Figure 5:**
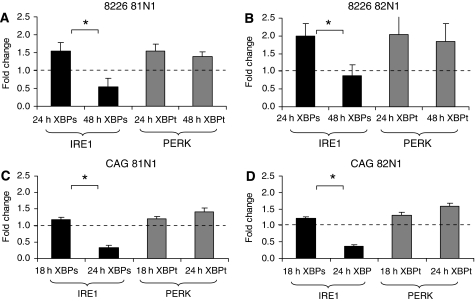
Fate of UPR signalling. Temporary increase in XBP1s (indicative of IRE1 pathway) and continued elevation of XBP1t (indicative of PERK pathway) in RPMI 8226 81N1 (**A**) 82N1 (**B**), and CAG 81N1 (**C**) and 82N1 (**D**) cells are presented. X-box binding protein spliced and total were detected by semi-quantitative RT–PCR. Expression levels of XBP1s and XBP1t were normalised to *β*-actin, fold changes were calculated relative to mock and expressed as mean±s.e. of at least three separate experiments. Statistically significant differences (^*^P<0.05) are depicted.

**Figure 6:**
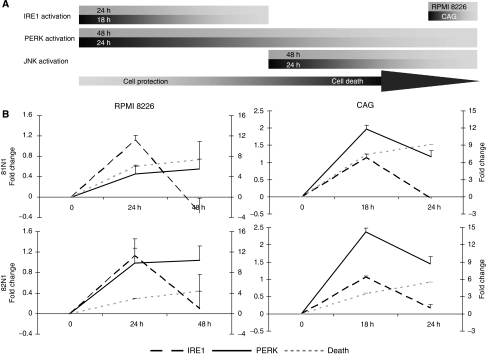
Timeline of UPR signalling and cell death. The figure presents the activation sequence of IRE1, PERK, JNK, and cell death of CD81N1/CD82N1-transfected RPMI 8226 and CAG MM cell lines. Inositol-requiring 1 and PERK were determined by semi-quantitative RT–PCR of XBP1s and total XBP1, respectively; JNK activation was deduced from rescue of tetraspanin-transfected cells (relative mock) by SP600125; and cell death was analysed by FACS based on PI exclusion of eGFP^+^ cells. The **A** panel is a schematic presentation and the **B** panel depicts mean fold change±s.e. of IRE1, PERK, and cell death (depicted in the legend under the graphs) relative to mock at different time points (x-axis). Significant elevation of IRE1 and PERK were at 24 h/18 h post-transfection in RPMI 8226 and CAG MM cells, respectively (*P*<0.05). As time progressed (48 h/24 h post-transfection in RPMI 8226 and CAG cells, respectively), it can be determined that, although IRE1 splicing of XBP1 was diminished, the PERK-regulated expression of total XBP1 was maintained. At least three separate experiments were conducted at each time point.

The feasibility of our findings is further confirmed by the timeline of UPR signalling and ensuing death (Figures 5 and 6), which is compatible with the model presented recently by Lin *et al* (2007). Lin and co-authors presented that the initial combined activation of IRE1, PERK, and ATF6 produces cytoprotective outputs (reduced translation, enhanced ER protein folding capacity), which provide a ‘window of opportunity’ for cells to readjust their ER to cope with stress. If these steps fail to re-establish homoeostasis, IRE1 signalling followed by ATF6 signalling is attenuated, creating an imbalance in which an unchecked PERK pro-apoptotic output channels the cell towards its termination (Lin *et al*, 2007). Figure 5 shows a temporary increase in XBP1s (*P*<0.05) and continued elevation of XBP1t in CD81N1/CD82N1-transfected MM cell lines. In Figure 6, we present a sequential alignment of UPR markers with cell fate. Inositol-requiring 1 and PERK activation was determined 24/18 h after tetraspanins’ transfection in RPMI 8226 and CAG cells, respectively. At 48/24 h post-transfection, PERK activation was still maintained in RPMI 8226 and CAG cells, respectively, whereas IRE1 activity had already decreased. At this time point, we also determined JNK activation and showed significant cell death. In addition, pJNK levels were not elevated at earlier time points (24 h in RPMI 8226 and 18 h in CAG cells) and, therefore, are in accordance with its activation in IRE1 progression and onset of cell death (Figure 6 and data not shown).

## Discussion

The primordial function of autophagy has long been recognised, but its significance in multiple cellular roles and various pathological conditions, including cancer, is only now being revealed (Gozuacik and Kimchi, 2007; Lerena *et al*, 2008; Mizushima *et al*, 2008). In this study, we showed that MM cell lines rely on autophagy for their survival and proliferation under normal culture circumstances. This was not evident in cell lines of other origins. The propensity of MM cells for increased autophagic activity is consistent with the published data that established their heightened ER-stress levels and the functional relationship between the two states (Cenci and Sitia, 2007). Despite the survival-promoting role of the basal autophagy, which we determined, it is well recognised that elevated and/or prolonged autophagy can culminate in cell death (Gozuacik and Kimchi, 2007; Galluzzi *et al*, 2008), a prospect of great significance in the death of resistant myeloma cells.

In this study, we establish that autophagic death is an achievable target in MM cell lines. We showed that tetraspanins induced ER-stress, manifested by activation of UPR pathways, increased autophagy, and eventually, cell death. Moreover, chronological alignment of UPR pathways’ expression, shutdown, and ensuing cell death is in agreement with the mechanism of cell fate regulation allocated with the UPR (Lin *et al*, 2007). The achievability of autophagic death in MM is strengthened by a recent study that reported that inhibition of p27 expression in MM caused death by autophagy (Chen *et al*, 2008). Yet, in this study, death was preceded by cell cycle arrest, whereas our experimental setting displayed no such effect (Tohami *et al*, 2007). Previously, we showed that the CD81N1/CD82N1 overexpression attenuated AKT activity and activated FoxO transcription factors (Lishner *et al*, 2008). In this project we again showed the involvement of JNK activity in the fate of transfected cells (Lishner *et al*, 2008). Taken together, these findings are compatible with a recent publication that depicted a role for FoxO1 in the death of ER-stressed macrophages (Senokuchi *et al*, 2008). Interestingly, Senokuchi and colleagues showed that FoxOs failed to induce cell death in the absence of ER-stress. The involvement of JNK in ER-stress response, as well as in the regulation of FoxO proteins has been described extensively (Ogata *et al*, 2006; Huang and Tindall, 2007; Lim *et al*, 2009).

The connection of tetraspanin circuitry with ER-stress and autophagy is novel and provides a direct link between the cancer microenvironment and fundamental cellular functions. In particular, positioning the tetraspanins up-stream of ER–Golgi homoeostasis underscores the significance of the integration of metabolic, environmental, and mitogenic cues to MM survival.

The biological validity of our experimental system is substantiated by multiple controls. In both the current and in previous (Tohami *et al*, 2007; Lishner *et al*, 2008) studies, we showed that cell death, as well as the activation of UPR pathways, can be determined only in MM cell lines transfected with CD81N1 and CD82N1 eGFP fusion vectors and not with the empty eGFP construct (mock) or with the CD81C1 and CD82C1 oriented fusion plasmids (Tohami *et al*, 2007). Taken together, our results can be interpreted as presenting specific signals initiated by the cloned tetraspanins (in their N1 conformation). Concurrent with this line of thought, studies underway in our laboratory are examining the possible involvement of phosphatidylinositol 4 kinase type II (PI4KII*α*/*β*) by direct or indirect (through other tetraspanin members) association with CD81N1/CD82N1. Phosphatidylinositol 4 kinase type II-*α* is a particularly attractive target because it is one of the few signalling molecules known to bind to tetraspanins and it is critically important for ER–Golgi homoeostasis. Moreover, its product, the phosphatidyl inositol-4-phosphate, functions as a docking/binding domain for multiple proteins (Weixel *et al*, 2005; Matteis and D’Angelo, 2007). The differential association of CD81 (binding) and CD82 (non-binding) (Yauch and Hemler, 2000) to PI4KII and the presence of CD81 in RPMI 8226 cells may result in diverse tetraspanin microdomains (qualitatively and quantitatively).

Our results can also be viewed from a totally different perspective. It is possible that ER-stress and autophagy death are instigated by the sheer burden of protein synthesis and trafficking, regardless of any specific signals that may originate from the tetraspanin fusion protein. It should also be taken into account that CD81/CD82 eGFP fusion proteins are regulated by the powerful CMV promoter, and that MM cells are already in a sensitised state of elevated protein synthesis and increased IRE/XBP1 expression. The differences between the pEGFP-N1-oriented plasmids and the empty or pEGFP-C1-oriented cloned tetraspanins may be attributed to decreased efficiency of folding and/or trafficking. If so, the transfected proteins would accumulate in the ER and eventually cause stress. Proof-of-concept will indicate that the mere burdening of ER–Golgi function may present a therapeutic target in protein-secreting cells such as myeloma. The effect of CD81N1 and CD82N1 on protein synthesis in the transfected MM cell lines is currently under investigation in our laboratory.

Targeting protein synthesis and secretion in MM has been addressed previously (Carew *et al*, 2006; Nakamura *et al*, 2006; Davenport *et al*, 2007; Patterson *et al*, 2008). Several drugs abrogating ER–Golgi stability have been used *in vitro* to induce MM cell death. In addition, a recent publication reported that the proteosome inhibitor, Bortezomib, used clinically causes protein accumulation in the ER by blocking ERAD as one of its action modes (McCloskey *et al*, 2008). Our findings suggest the possibility that ER-stress may be achieved by increased protein synthesis and interestingly, this may be the path of least resistance, because it is in sync with built-in MM characteristics, hence the ‘Achilles’ heel’ of these cells. Future studies will be needed to determine effective ways to induce catastrophically elevated and clinically achievable protein synthesis for therapeutic purposes.

## Figures and Tables

**Table 1:** Summary of CD81N1/CD82N1-induced effects in MM cell lines

**Model**		**UPR**		**Cell death mode**		**Cell death rescue**	
**Cell line**	**Tetraspanin**	**IRE**	**PERK**	**Necrosis (PI^+^cells↑)**	**Autophagy (LC3II↑)**	**3MA**	**MAPK (JNK/ERK) inhibition**
8226	81N1	√	√	√	√	√	√
8226	82N1	√	√	√	√	√	√
CAG	81N1	√	√	√	√	√	√
CAG	82N1	√	√	√	×	×	×

Abbreviations: ERK=extracellular signal-regulated kinase; IRE=inositol-requiring 1; JNK=c-Jun *N*-terminal kinases; MA=methyladenine; MAPK=mitogen-activated protein kinase; MM=multiple myeloma; PERK=protein kinase-like ER kinase; PI=propidium iodide; UPR=unfolded protein response.√

, An effect was found. × , no effect was evident. (Detailed in the results).
